# Supporting participation in paid work of cancer survivors and their partners in the Netherlands: protocol of the SusTained Employability in cancer Patients and their partnerS (STEPS) multi-centre randomized controlled trial and cohort study

**DOI:** 10.1186/s12889-021-11865-8

**Published:** 2021-10-12

**Authors:** Amber D. Zegers, Pieter Coenen, Ute Bültmann, Valesca Retèl, Jacobien M. Kieffer, Allard J. van der Beek, Saskia F. A. Duijts

**Affiliations:** 1 Department of Public and Occupational Health, Amsterdam Public Health Research Institute, Amsterdam UMC, Vrije Universiteit Amsterdam, location VUmc, van der Boechorststraat 7, 1081 BT Amsterdam, The Netherlands; 2 University of Groningen, University Medical Center Groningen, Department of Health Sciences, Community and Occupational Medicine, Groningen, the Netherlands; 3 The Netherlands Cancer Institute, Department of Psychosocial Research and Epidemiology, Amsterdam, the Netherlands; 4 The Netherlands Comprehensive Cancer Organization (IKNL), Research & Development, Utrecht, the Netherlands

**Keywords:** Cancer survivors, Spouses, Vocational rehabilitation, Clinical trial protocol, Cohort study

## Abstract

**Background:**

Many cancer survivors experience physical and/or psychosocial problems affecting return to work (RTW) and work retention. Current interventions on RTW lack evidence regarding effectiveness, while interventions for work retention are missing. Partners of cancer survivors may also experience work- and health-related outcomes; yet, these consequences are not well understood. Here, the protocol of the STEPS study is described. The study aims are to: 1) evaluate the (cost-)effectiveness of a rehabilitation program for RTW and work retention in cancer survivors, and 2) assess health- and work-related outcomes among cancer survivors’ partners.

**Methods:**

In a multicentre Randomized Controlled Trial (RCT), 236 working-age cancer survivors with an employment contract will be randomly allocated to a usual care group or an intervention group receiving a multidisciplinary rehabilitation program, combining occupational therapy facilitating work retention (e.g., energy management and self-efficacy training) and reintegration consultation addressing work-related issues (e.g., RTW planning and discussing workplace or task modifications with the supervisor). Alongside the RCT, a prospective cohort study will be conducted among cancer survivors’ partners (*n* = 267). Participants in the RCT and cohort study will be asked to complete questionnaires at baseline, and after six and 12 months, assessing work- and health-related outcomes. Generalized estimating equations will be used to assess intervention’s effectiveness, compared to usual care, regarding primary (i.e., working hours per week) and secondary outcomes. Also economic and process evaluations will be performed. For the cohort study, logistic or linear regression modelling will be applied assessing work- and health-related outcomes (primary outcome: working hours) of cancer survivors’ partners, and what factors predict these outcomes.

**Results:**

The study is planned to start in September 2021; results are expected in 2023.

**Conclusion:**

Compared to usual care, the STEPS intervention is hypothesized to be (cost-)effective and the intervention could be a valuable addition to standard care helping cancer survivors to sustain employment. Further, it is expected that living with a cancer survivor has a substantial impact on work and health of partners, while specific groups of partners that are at particular risk for this impact are likely to be identified.

**Trial registration:**

Dutch Trial Register (NTR;NL9094; 15-12-2020).

**Supplementary Information:**

The online version contains supplementary material available at 10.1186/s12889-021-11865-8.

## Background

In the Netherlands, roughly 118,500 individuals are newly diagnosed with cancer each year (https://www.iknl.nl/en/ncr), with approximately 40–50% being of working age. Although 64% will return to work (RTW) after their diagnosis [1], many cancer survivors experience long-term psychosocial (e.g., feelings of depression, anxiety, uselessness and loneliness), physical (e.g., fatigue, pain, nausea, menopausal symptoms and movement limitations), and/or cognitive difficulties (e.g., changes in ability to multitask and working memory), possibly affecting their working lives [2, 3]. Despite these difficulties, work-related care is often not or not systematically provided to cancer survivors [4]. In this study we define cancer survivorship as ‘a process that begins at the moment of diagnosis and continues through the balance of life’ [5].

In the past decades, various interventions have been developed or suggested to support RTW in cancer survivors, including single-component psycho-educational, physical, and medical interventions, and multi-component interventions, such as physical exercise, biofeedback-assisted behavioural training, patient consultation and education, and/or vocational consultation [6]. To date, only weak evidence is available stating that multi-component interventions improve (time to) RTW in cancer survivors, with no evidence supporting the effectiveness of single-component interventions on RTW. Moreover, the majority of evidence is derived from studies conducted in breast cancer survivors, which poses a problem for generalization to other cancer diagnoses and treatment sequelae. Further, while most studies have focussed on RTW after cancer, only few have addressed retention of paid employment beyond RTW. In a recent study among Japanese male cancer survivors, 80% had successfully retained work 1 year after RTW [7]. However, this percentage dropped to 49% after 5 years. Previous studies showed that work retention rates vary significantly with cancer type, with the highest drop-out rates after initial RTW in survivors of lung, liver, pancreas, and oesophageal cancer [7]. Findings from a systematic review showed that 73% of long-term cancer survivors that were working at time of diagnosis managed to retain work 2 years after their diagnosis [8]. Yet, a recent study has shown that many cancer survivors wish to receive work-related care early after diagnosis in the hospital setting, with only few cancer survivors actually receiving such early care [4]. These studies illustrate the difficulty of RTW and retaining paid employment, beyond initial reintegration at the workplace, for cancer survivors. However, few early intervention studies have been conducted to prevent adverse work outcomes and support work retention in cancer survivors [1, 9].

Recently, sustained employment (i.e., RTW and work retention) has been conceptualized as a health *behaviour*, i.e., something a person does or does not do, and that is, to some degree, changeable [10]. Behavioural change models, such as the Stages of Change model [11], have been applied to the development of lifestyle interventions in cancer survivors (e.g., for smoking cessation [12]). A relatively new phenomenon in the scientific community is the application of this model to interventions for sustained employment in cancer survivors [13, 14]. It is theorized that cancer survivors move through several stages of behavioural change regarding RTW and work retention (i.e., pre-contemplation, contemplation, preparation: self-evaluative, preparation: behavioural, uncertain and proactive maintenance) in a non-linear manner (i.e., they can skip stages or lapse back to prior stages) [10]. By identifying a cancer survivor’s behavioural change stage, interventions to support sustained employment can be tailored to better fit the individual’s circumstances [10]. Interventions can therefore be tailored to cancer survivors ranging from those who are starting to consider RTW to those who have already returned to work and are looking to retain work. Providing cancer survivors with a tangible overview of their current stage within the employment trajectory might create a sense of acknowledgment and normalcy, and thereby encourage them to see the bigger picture and formulate work-related goals. The concept of readiness for RTW (RRTW) [15] builds on the Stages of Change model [11] to describe motivational factors contributing to and maintaining behaviour change in the context of sustained employment [15]. Prior research has shown that cancer survivors view the determination of ‘work readiness’ as an important step in the RTW process, and would like to receive guidance in this from health care professionals early on in their illness-trajectory [16]. Recently, Nielsen and colleagues conducted a qualitative study amongst Danish cancer survivors and, again, found that ‘becoming ready’ is a process cancer survivors would like to receive guidance in [17]. To date, however, the application of the Stages of Change model [11] in an intervention to support RTW or work retention has been trialled in some studies (e.g. [18]), but has not been thoroughly explored in cancer survivors.

Contrary to the work-related outcomes of cancer survivors, relatively little is known about the health- and work-related outcomes of cancer survivors’ *partners* (i.e. those who are married to or cohabit with a cancer survivor). It has been reported that the fear of losing their significant other can cause an increase in distress, anxiety and depressions among cancer survivors’ partners [19]. Veenstra and colleagues found that partners of breast cancer survivors reported a negative impact on their financial status, employment status, and health care insurance [20]. These feelings were more pronounced if partners had the financial responsibility for their family, and the majority of partners reported that it was very important to stay in their current job to maintain health insurance. With different social and health care systems, such effects may differ between countries. For example, in the Netherlands, one is obliged to have health care insurance, which is unrelated to having paid employment. There is, however, both globally and in the Netherlands, a lack of knowledge on the work- and health-related outcomes of partners of cancer survivors.

From the above it follows that: 1) work is important for many cancer survivors and their partners, 2) interventions to support RTW in cancer survivors showed limited effectiveness so far, 3) work retention in cancer survivors (beyond initial RTW) is often underrepresented in studies, 4) the Stages of Change model appears a promising angle for interventions aimed at supporting sustained employment of cancer survivors, and 5) little is known about the work- and health-related outcomes of partners of cancer survivors. Therefore, based on identified limitations, the SusTained Employability in cancer Patients and their partnerS (STEPS) study was developed, consisting of:

- a multi-centre Randomized Controlled Trial (RCT) to assess the (cost-)effectiveness of the STEPS intervention to support sustained employability (RTW and work retention) in cancer survivors. STEPS is a multidisciplinary intervention that combines the expertise of occupational therapists and reintegration consultants to support RTW and work retention in cancer survivors. STEPS is offered relatively early (i.e., 3–18 months) post-diagnosis to cancer survivors who had an employment contract at time of diagnosis and who are at work or (partly) on sick leave at the time of inclusion.
- a prospective cohort study to assess work- and health-related outcomes in partners of cancer survivors and identify what factors predict these outcomes.

In this paper the rationale for and protocol of the STEPS study will be described.

## Methods

### Design and setting

**Fig. 1:**
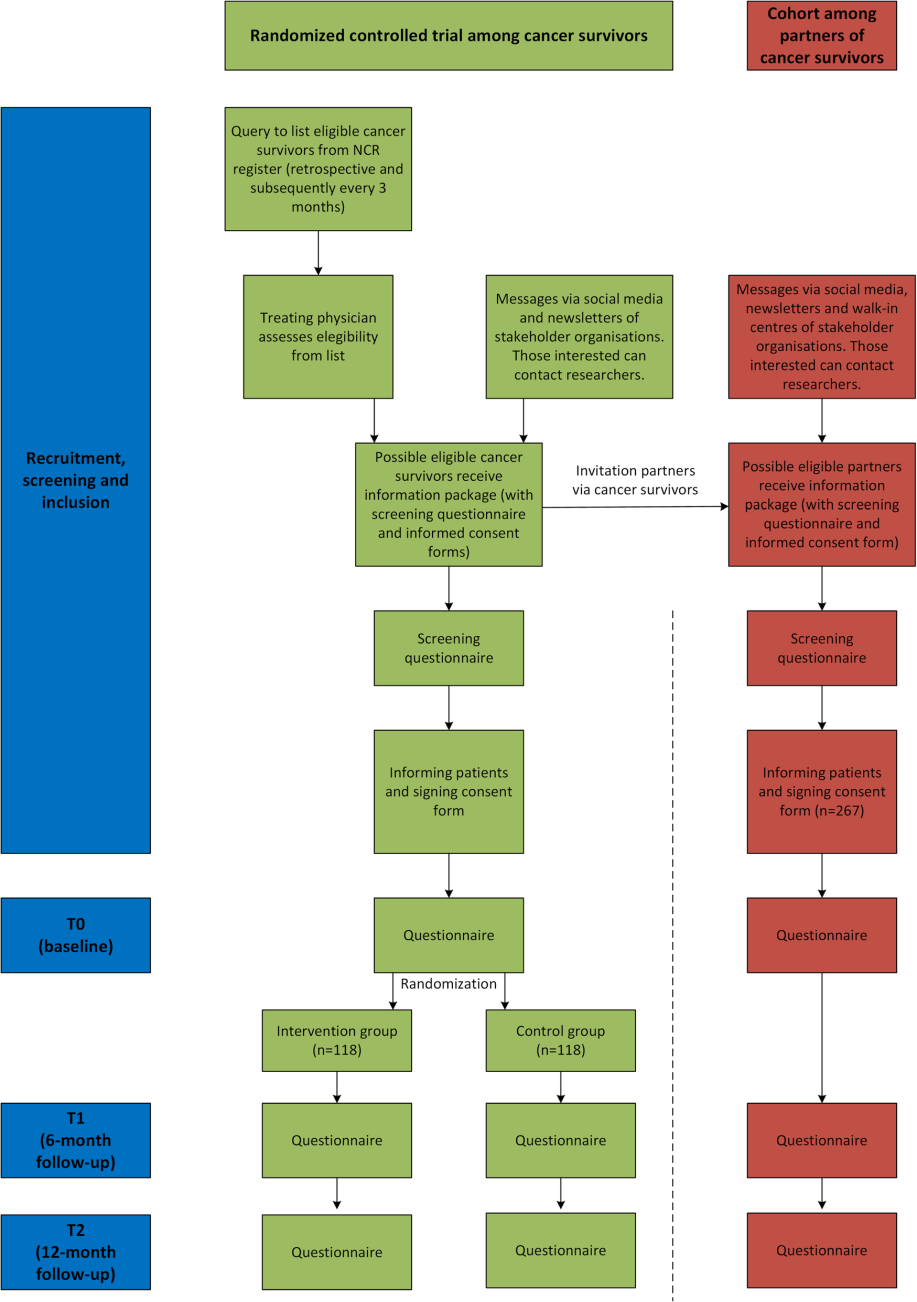
Overview of the study procedures, including recruitment, screening and inclusion. NCR = Netherlands Cancer Registry

The STEPS study consists of a multi-centre RCT to assess the (cost-)effectiveness of an intervention to support RTW and work retention, and a prospective cohort study into the work- and health-related outcomes of partners of cancer survivors. See Fig. 1 for a full overview of the study procedures, which are described in more detail below. The study will be executed by researchers of Amsterdam UMC (location VUmc) and has been approved by its Medical Ethical Committee (reference no. **2020.055).** All procedures will be in accordance with the ethical standards of this local ethics committee and with the Helsinki Declaration of 1975, as revised in 2000. The study has been registered at the Dutch Trial Register (registration no. NTR NL9094; registration date 15-12-2020; https://www.trialregister.nl/trial/9094). Substantial modifications to the protocol will be registered in the trial register (of which an audit trial will be held) and will be disclosed to the local Medical Ethical Committee.

#### RCT among cancer survivors

This protocol was written according to standard protocol items for clinical trials (SPIRIT) guidelines [21]. The (cost-)effectiveness of the STEPS rehabilitation program will be tested in a multi-centre, two-armed RCT among cancer survivors who are currently receiving, or have recently completed, oncological treatment in one of the participating academic and general hospitals throughout the Netherlands (Supplementary file 1). Eligible cancer survivors will be randomly allocated to the intervention group (receiving the STEPS program) or control group (receiving usual care) and will be asked to complete questionnaires prior to randomization (T0), and after six (T1) and 12 months (T2) follow-up.

#### Cohort study among partners of cancer survivors

In the cohort study, partners of cancer survivors (among others, partners of cancer survivors who participate in the aforementioned RCT) will be recruited. All included partners will be asked to complete questionnaires regarding their work and health at baseline (T0), and at six (T1) and 12 months (T2) follow-up.

### Participants and recruitment

#### RCT among cancer survivors

Cancer survivors of working age (18–63 years at time of diagnosis), with histologically confirmed cancer and a life expectancy of more than 1 year will be recruited. At study entry, eligible cancer survivors have a fixed or temporary employment contract, with at least 6 months left of their contract, and a history of paid work for at least 1 year prior to diagnosis (with ≥8 contracted working hours per week). Cancer survivors can both be at work or (partly) on sick leave when entering the study. Cancer survivors will be recruited 3–18 months post-diagnosis. The cut-off for the maximum time post-diagnosis was set at 18 months for cancer survivors to be able to complete the intervention (with a maximum duration of 6 months) before a potential work disability assessment. According to Dutch legislation, work disability assessments take place after two years of sick leave to determine whether someone is disabled for work or not and may receive work disability benefits or not. Regardless of the outcome of the work disability assessment, employers are allowed to terminate the employment contract after 2 years of sick leave. Moreover, cancer survivors will be eligible for participation in STEPS if they are, or have been, treated with at least radiotherapy and/or chemotherapy. Previous research has shown that cancer survivors who received complex treatments, i.e., chemotherapy, radiotherapy, or a combination thereof, indicated lower work ability than cancer survivors who were treated with surgery alone [22]. Cancer survivors with additional treatment modalities, besides radiotherapy and/or chemotherapy, will be eligible for participation as well.

Cancer survivors will be excluded if their treating physicians consider work unfeasible, if cancer survivors have serious cognitive or psychiatric problems, or other comorbidities that would preclude them from participating in the intervention program, and/or if cancer survivors lack basic proficiency in Dutch. Cancer survivors participating in concurrent studies or rehabilitation programs aimed at sustained employment, or cancer survivors who refuse the involvement of their employer in the STEPS intervention will also be excluded.

#### Cohort study among partners of cancer survivors

Partners are defined as being married to or cohabiting with a cancer survivor who participates in the aforementioned RCT. Cancer survivors and partners of survivors will be recruited independently of each other (and will not be jointly analysed). This means that both partners of cancer survivors who do and do not participate in the RCT will be eligible to participate in the cohort study. Partners of cancer survivors who have had a cancer diagnosis no more than 24 months ago and who have a life expectancy of at least 1 year, are eligible. Partners should be registered at the same address as the cancer survivor at least 1 year pre-diagnosis. Moreover, eligible partners should be between 18 and 65 years of age, have a fixed or a temporary employment contract at the time of diagnosis of the cancer survivors, and a history of paid work for at least 1 year prior to diagnosis (with ≥8 contracted working hours per week). Partners of cancer survivors can both be at work or (partly) on sick leave when entering the study. Partners will be excluded in case of self-reported serious cognitive or psychiatric problems that would prevent them from completing the questionnaires, and/or in case they are unable to understand and complete questionnaires in Dutch.

### Study procedures

#### RCT among cancer survivors

The main route of identification of potentially eligible cancer survivors is through the Netherlands Cancer Registry (NCR). Data managers of the NCR will develop a query based on (a selection of) aforementioned inclusion/exclusion criteria supplied by the researchers. The query will be performed once retrospectively (3–18 months), and prospectively every 3 months for 1 year. The list of potentially eligible cancer survivors will be sent to the involved medical specialist of a participating hospital, who will check whether these cancer survivors are indeed potentially eligible for participation. All potentially eligible cancer survivors will receive an information package with an invitation letter on behalf of the involved medical specialist of the participating hospital, an information brochure, a screening informed consent form, a screening questionnaire and a non-response answer card. If interested, cancer survivors will be asked to sign the screening informed consent form, complete the screening questionnaire and send these documents to the researchers. Cancer survivors will be asked to take a legally mandatory reflection period of at least 1 week before agreeing to participate. Survivors who are not interested in participating can complete a short non-response answer card on which they can indicate their reasons of non-response.

Cancer survivors who meet the inclusion criteria based on the screening questionnaire, will receive a telephone call from a research assistant during which they will be further informed about the project, in- and exclusion criteria will be confirmed, and they will have the opportunity to ask questions. If the cancer survivors’ eligibility is confirmed, they will be asked to sign a second informed consent form for study participation, and subsequently to complete the baseline questionnaire. Upon return of the completed baseline questionnaire, randomisation will take place.

Next to identification via the NCR and recruitment through hospitals, cancer survivors will be recruited through social media channels and (e-mail) messages of the Dutch Federation for Cancer Patient Organizations (NFK), Kanker.nl (a Dutch platform for cancer survivors), and psychosocial oncology walk-in centres (Instellingen PsychoSociale Oncologie; IPSO). Cancer survivors can contact the research team if they wish to receive aforementioned information package. This package will then be sent via post, after which the recruitment procedure is identical to that described above.

#### Cohort study among partners of cancer survivors

Partners of cancer survivors will be invited to participate in the cohort study through the cancer survivors who received an information package regarding the RCT. An invitation letter, information brochure, informed consent form, screening questionnaire and non-response answer card will be included in a separate package, which the cancer survivor (if applicable) may hand over to his/her partner. Partners will be asked to take a legally mandatory reflection period of at least 1 week before agreeing to participate. If a partner is interested, (s) he is asked to sign the informed consent form and complete the screening questionnaire, based on which eligibility will be checked. Upon request partners are given the opportunity to ask questions about the study in a telephone call. Partners who are not interested in participating can complete a short non-response answer card on which they can indicate their reasons of non-response.

Additional recruitment pathways (similar to the one for cancer survivors described above) consist of social media and e-mail messages through NFK, Kanker.nl, and IPSO walk-in centres.

### Sample size calculation

#### RCT among cancer survivors

The RCT sample size calculation is based on the primary self-reported outcome measure, i.e., working hours per week. Based on previous research [23], a standard deviation of 13 h per week was assumed, and a between group (intervention versus control) effect of 5.5 h per week is expected. Given this effect size, with a power of 80% and level of statistical significance set at *p*-value< 0.05, the sample size required is 88 cancer survivors in each group (176 in total). In this study 236 cancer survivors will be recruited, to allow for an attrition rate of approximately 25% (i.e., cancer survivors who discontinue participation in the study entirely, including failure to complete the follow-up questionnaires) and to enable subgroup analyses.

#### Cohort study among partners of cancer survivors

Because of the lack of literature on work- and health-related outcomes of partners of cancer survivors, the sample size calculation is based on the secondary objective of the cohort study, i.e., identifying factors to predict partner’s work- and health-related outcomes. A generally accepted formula was used to estimate the sample size [24], i.e., *n* = 10 observations per variable in the smallest outcome group. Hence, to generate the prediction model with a maximum of 10 variables a sample size of at least *n* = 100 in the smallest outcome group is required. As the ratio of partners with positive and negative work- and health-related outcomes is expected to be 50:50, a total sample size of *n* = 200 is required. About 267 partners will be recruited, to allow for an attrition rate of approximately 25%.

### Randomization

After signing the informed consent form for study participation and completion of the baseline questionnaire, randomization will be performed by the study leader (ADZ) or a research assistant using Castor EDC (https://www.castoredc.com), a Good Clinical Practice (GCP-)approved online tool, to divide cancer survivors in either the STEPS intervention or usual care control group. Variable block randomization will be applied with stratification per recruiting hospital. In Castor EDC, allocation sequence is concealed. However, due to the nature of the intervention, neither cancer survivors nor those who deliver the intervention or analyse the results will be blinded to the randomization. All cancer survivors randomized to one of the two trial arms will be considered to be included in the study, and will thus be followed during the follow-up period.

### STEPS intervention

STEPS is a multidisciplinary intervention based on the Stages of Change, and combines work-related occupational therapy and reintegration consultation, to support cancer survivors in their RTW and work retention. The STEPS intervention program was designed both bottom-up (i.e., theory-driven, using the Stages of Change model [11]) and top-down (i.e., by interviews with experts and cancer survivors, as described in more detail elsewhere (Zegers AD, Coenen P, Bültmann, van de Poll-Franse LV, van der Beek AJ, Duijts SFA. Tailoring work participation support for cancer survivors using the Readiness for Return to Work scale: perspectives and opinions of (health care) professionals and cancer survivors. In preparation.)). The intervention will be delivered by both occupational therapists and reintegration consultants, who tailor the intervention content according to the Stages of Change as measured by the RRTW. Occupational therapists are working at the outpatient clinics of participating hospitals. Most occupational therapists involved in the delivery of STEPS have experience with work-related guidance of cancer survivors. Those who do not, have experience with providing other aspects of occupational therapy to cancer survivors. All involved occupational therapists received a half day STEPS intervention training and will participate in peer intervision sessions throughout the intervention period. Involved reintegration consultants in STEPS are employed at a private reintegration consultancy, specialized and experienced in work-related support of cancer survivors. Reintegration consultants were trained in the STEPS intervention content as well. The content of the STEPS intervention has been described in the 89-page STEPS intervention handbook (in Dutch, available in PDF upon request). The intervention was designed to be delivered face-to-face, but will also be available remotely (i.e., using video conferencing or telephone meetings) if necessary (e.g., in case of COVID-19 regulations).

**Fig. 2:**
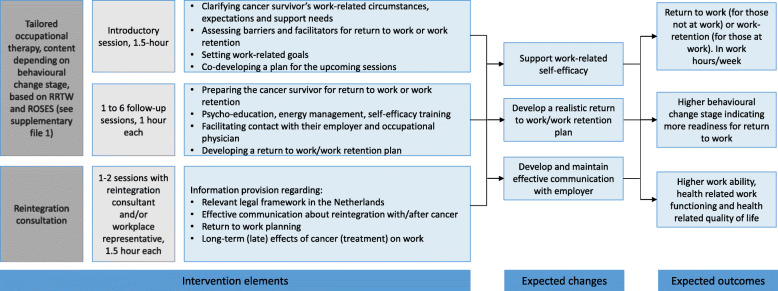
Logic model of the STEPS intervention elements, expected changes and outcomes

See Fig. 2 for a logic model of the STEPS intervention elements, and Supplementary file 2 for an overview of the intervention components per behavioural change stage. The STEPS intervention has a maximum duration of 6 months, during which cancer survivors receive a minimum of three and a maximum of nine sessions, depending on their work-related support needs. STEPS is aimed at supporting cancer survivors’ work-related self-efficacy during their RTW or work retention process, and to encourage them to develop and maintain effective communication with their employer. During the STEPS intervention, the cancer survivors’ behavioural change stage will be monitored continuously and the intervention will be tailored to the specific stage the survivor is in. To do so, cancer survivors will be asked to complete a brief questionnaire (i.e., a Dutch version [25] of the Readiness for Return To Work - RRTW [15]) and an abbreviated version of the Return to Work Obstacles and Self-Efficacy Scale - ROSES [26]) prior to each session with the occupational therapist. Based on the outcomes of the RRTW questionnaire, participants will be categorised according to one of the six stages of change (pre-contemplation, contemplation, preparation: self-evaluative, preparation: behavioural, uncertain and proactive maintenance). Within each of these stages, participants will furthermore be given a traffic light designation (i.e. red, orange or green), based on the outcomes of the ROSES questionnaire, depicting potential barriers and facilitators regarding sustained employment and their overall progress within the intervention. Based on this categorisation the occupational therapist can, using the STEPS handbook, tailor the intervention provided (see Supplementary File 2).

The intervention includes the following elements:

1. Introductory session (1.5 h duration) that will take place with the occupational therapist and the cancer survivor. The session is aimed at clarifying the cancer survivor’s work-related circumstances, expectations, and support needs, including barriers and facilitators for RTW or work retention (i.e., using the RRTW [15]) and ROSES [26] questionnaires), setting work-related goals, and co-developing a plan for the upcoming sessions.
2. A minimum of one and a maximum of six one-on-one sessions (1 h duration each) with the occupational therapist and the cancer survivor. These sessions are aimed at preparing the cancer survivor for RTW or work retention, using psycho-education, energy management, self-efficacy training, facilitating contact with their employer and occupational physician, and developing and trying out a RTW/work retention plan.
3. A minimum of one and a maximum of two sessions (1.5 h duration each) with the reintegration consultant and the cancer survivor. These sessions are aimed at providing information regarding the relevant legal framework in the Netherlands, effective communication about reintegration with/after cancer, RTW planning, or the long-term effects of cancer (treatment) on work. These information sessions can take place one-on-one, or together with a relevant person from the cancer survivor’s workplace (e.g., direct supervisor or human resource officer).

### Care as usual

Participants in the control group will receive usual care regarding sustained employment, the content and availability of which will vary per participating hospital. In the Netherlands, there is no structured provision of work-related care for cancer survivors in hospitals, and we have shown before that the extent to which such care is provided to cancer survivors is therefore limited [4]. Nonetheless, work-related care is sometimes delivered as part of the care provided by various health care providers including occupational therapists, social workers, rehabilitation care practitioners or physiotherapists.

### Measurements

Baseline and follow-up questionnaires for both the RCT and cohort study will be digitally administered using Castor EDC (https://www.castoredc.com). Questionnaires can be accessed through a link that will be sent via e-mail. Participants who have expressed a preference for hardcopy versions of the questionnaires will receive them at their home address. Participants will receive one reminder for each questionnaire, 2 weeks after the questionnaire has been sent. If participants do not return the six-month follow-up questionnaire, and unless they have withdrawn their participation from the study, they will receive the 12-month follow-up questionnaire. Table 1 provides an overview of all measurements incorporated in the questionnaires (primary and secondary outcomes and additional measurements; for cancer survivors and partners). Table 2 shows how all constructs will be operationalized, measured, and processed for further analyses, including references to individual questionnaires.

**Table 1:** Overview of variables that will be measured in the STEPS randomized controlled trial (RCT) among cancer survivors and the cohort study among partners of cancer survivors

Category	Variable	RCT among cancer survivors				Cohort study among partners of cancer survivors			
		T0 (Baseline)	T1 (6-month)	T2 (12-month)	Variable^a^	T0 (Baseline)	T1 (6-month)	T2 (12-month)	Variable^a^
Work outcomes	Working hours per week	X	X	X	P	X	X	X	P
	Change in working hours (%)	X	X	X	S	X	X	X	S
	Employment status	X	X	X	S	X	X	X	S
	Time to return to work	X	X	X	S^b^				–
	Sick leave	X	X	X	S	X	X	X	S
	Readiness for return to work	X	X	X	S				–
	Work ability	X	X	X	S				A
Health outcomes	Health-related work functioning	X	X	X	S^c^	X	X	X	S
	Health-related quality of life	X	X	X	S	X	X	X	P
	Caregiver burden				–	X	X	X	S
	Depression	X			A	X	X	X	S
Sociodemographic	Age, gender	X			A, M	X	X		A
	Marital status	X			A	X	X		A
	Children living at home	X			A	X	X		A
	Education and income	X			A	X	X		A
	Breadwinner status	X			A	X	X		A
Medical	Cancer site	X			A, M	X	X		A^d^
	Time of diagnosis	X			A, M	X	X		A^d^
	Received and future treatment(s)	X			A, M	X	X		A^d^
	Cancer recurrence(s)	X			A	X	X		A^d^
Lifestyle	Smoking	X			A	X	X		A
	Alcohol consumption	X			A	X	X		A
	Physical activity	X			A	X	X		A
Health	Comorbidities	X			A	X	X		A
	Fatigue	X			A	X	X		A
Employment	Main tasks	X			A	X	X		A
	Years in current position	X			A	X	X		A
	Years of paid employment (and in current job)	X			A	X	X		A
	Contract type (fixed/temporary)	X			A	X	X		A
	Shift work	X			A	X	X		A
	Company size and sector	X			A	X	X		A
	Time since first day of sick leave	X			A				–
	Employment status partner (if applicable)	X			A				–
	(duration of) Caregiving leave				–	X	X		A
Work	Social support from supervisor/colleagues	X			A	X	X		A
	Job insecurity	X			A	X	X		A
	Need for recovery	X			A^c^	X	X		A
	Work accommodation	X			A^c^	X	X		A
	Impact of COVID-19 on work	X			A^c^	X	X		A
	Work attitude	X			A	X	X		A
	Self-efficacy	X			A	X	X		A
	Work-family balance	X			A^c^	X	X		A
	Work intention	X			A	X	X		A
	Fear of COVID-19	X			A	X	X		A
	Expectations regarding return to work	X			A				–
Process measures			X		P				–
Economic evaluation	Quality of life	X	X	X	E				–
	Health care consumption	X	X	X	E				
	Productivity loss	X	X	X	E				

^a^*P* Primary outcome variable, *S* Secondary outcome variable, *A* Additional variable, *M* Modifier, *E* Economic evaluation variables, *P* Process variables

^b^Only completed by participants who were on sick leave at baseline

^c^Only completed by participants who (partly) returned to work

^d^The partner will be asked regarding the medical status of the cancer survivor

**Table 2:** List of primary, secondary and additional variables and their assessment methods

		Instrument	# items	Rating scale	Operationalization
Work outcomes	Working hours per week	Asking participants for their current number of actual working hours	1	n/a	Continuous
	Change in working hours (%)	Asking participants for their current number of actual working hours and their contractual working hours at baseline.	1	n/a	Continuous (actual working hours at follow-up as a percentage of contractual working hours at baseline).
	Employment status	Asking participants whether they are at work or not at work	1	n/a	Dichotomous
	Time to return to work	Asking participants about their first day of returning to work, from which we will estimate the number of calendar days between the first day of sick leave and the first day of work, either fulltime or part-time, for at least 28 consecutive days without recurrence.	1	n/a	Continuous
	Sick leave	Asking participants about the number of days they have been on sick leave in the past 6 months	1	n/a	Continuous
	Readiness for return to work	The translated and adapted Dutch version [25] of the original RRTW questionnaire [15]. There are a number of items for each of the phases, with each item being scored on a 1 to 5 scale. Each RRTW stage will be scored by averaging all items within that stage. The stage with the highest summary score was considered the stage the participant was in. In case of a tie between stages, the lowest RRTW stage will be adopted. Based on this participants will be classified in any of the six RRTW phases.	21	6 stages	Ordinal
	Work ability	Single question of the Work Ability Index (WAI) [27], asking participants to estimate their current work ability compared with their lifetime best on 0 to 10 scale (0 = cannot work at all; 10 = best ever);	1	10-point scale	Continuous
Health outcomes	Health-related work functioning	The validated [28] 27-item Work Role Functioning Questionnaire (WRFQ) [29], distinguishing five different work domains: work scheduling demands, mental demands, social demands, physical demands, and output demands (range 0 to 100). Higher scores indicate better work functioning.	27	0 to 100	Continuous
	Health-related quality of life	For patients: the European Organization for Research and Treatment of Cancer Quality of Life Questionnaire – Core 30 (EORTC QLQ C-30) [30]). This 30-item list consists of five multi-item functional scales (i.e., physical, role, emotional, cognitive, and social), three multi-item symptom scales (i.e., fatigue, pain, and nausea and vomiting), six single-item (i.e., dyspnoea, insomnia, appetite loss, constipation, diarrhoea, and financial impact), and a two-item global health and quality of life scale, all with a scoring range from 0 to 100. A higher score on the functional and global health and quality of life scales indicates better quality of life, while on the symptom scales, a higher score indicates a higher level of symptom burden. A summary score will be generated, calculated as the mean of all combined scale scores, excluding financial impact and the global health and quality of life scale.	30	0 to 100	Continuous
		For partners: 12-item short-form health survey (SF-12) [31], expressed in t-score.	12	n/a	Continuous
	Caregiver burden	The ‘Ervaren Druk door Informele Zorg-plus’ (EDIZ) [32], consisting of 15 items, ranging from 0 to 1, higher scores indicate a higher caregiver burden	15	0 to 15	Continuous
	Depression	The Centre for Epidemiological Studies Depression Scale [33];, consisting of 20 items and a range of 0 to 60, a score of 16 and over indicates the possible presence of clinical depression	20	0 to 60	Dichotomous
Sociodemographic	Age	Asking participants about their age (in years)	1	n/a	Continuous
	Gender	Asking participants about their gender (male/female)	1	n/a	Dichotomous
	Marital status	Asking participants about their marital status with answer options: Not married, married without children, married with children, living together with partner, living together with partner and children, living together with others, single with children, divorced, widow/widower.	1	n/a	Categorical
	Children	Number of children (living at home)	2	n/a	Continuous
	Education	Asking participants about their highest education level completed with eight outcome options according to the Dutch education system.	1	n/a	Categorized in low, middle, high (according to International Standard Classification of Education (ISCED-97).
	Income	Asking participants about their annual income (before tax) with answer options: <€30.000, €30.000–€60.000, €60.000–€100.000, and > €100.000.	1	n/a	Categorical
	Breadwinner status	Asking participants about their breadwinner status with answer options: being sole breadwinner, not being breadwinner, being shared breadwinner with partner, don’t know	1	n/a	Categorical
	Financial necessity of work	Asking about financial necessity of work	1	n/	Dichotomous
Medical	Cancer site*	Asking participants about their cancer site with 17 answer options (being the most common cancer sites) and the answer option ‘other’	1	n/a	Categorical
	Time of diagnosis*	Asking participants for their time of diagnosis, which can be calculated in the time since diagnosis.	1	n/a	Continuous
	Treatment(s)*	Received and future treatment(s). E.g. surgery, radiation, chemotherapy, hormonal therapy, or combination, and medication	1	n/a	Categorical
	Cancer recurrence(s)*	Asking about recurrence of cancer in the follow-up questionnaires.	1	n/a	Dichotomous
Lifestyle	Smoking	Asking participants about their smoking status with answer options: smoker, ex-smoker, non-smoker.	1	n/a	Categorical
	Alcohol consumption	Asking participants about their alcohol consumption with answer options: Never, only at festive occasions, several times a month, once a week, several times a week, daily.	1	n/a	Categorical
	Physical activity	Asking participants about how many days a week they spend at least 30 min cycling, household activities, gardening or sports.	1	0 to 7	Continuous
Health	Comorbidities	14-item comorbidity questionnaire [34]. For partners this also includes a question about cancer.	14	0, 1–2 or ≥ 3 comorbidities	Categorical
	Fatigue	For cancer survivors: Functional Assessment of Chronic Illness – Fatigue scale (FACIT-F [35];). This 13-item questionnaire has a scoring range from 0 to 52. A higher score on this scale means less fatigue.	13	0 to 52	Continuous
		For partners: The ‘Checklist Individuele Spankracht’ (Checklist Individual Strength) that has been described in Dutch [36] and has been used on samples of workers before [37]. The scale consists of 20 items with outcome categories on a 1 to 7 scale. Summary scores (potentially using four subscales) range from 20 to 140, with higher scores indicating more fatigue.	20	20 to 140	Continuous
Employment	Main tasks	Asking participants whether they engage in a job with mainly mental, mainly physical or a combination of mental and physical tasks	1	n/a	Categorical
	Years in current position	Asking participants about how many years they have spent in the current job position regardless of current employer.	1	n/a	Continuous
	Years of paid employment	Asking participants about how many years they have spent working in general and at their current employer	1	n/a	Continuous
	Contract type	Asking participants about their contract type with four answer options: permanent contract, contract for fixed amount of time, secondment contract, contract through employment agency.	1	n/a	Categorical
	Shift work	Asking participants what sort of shifts they work in with answer options: day shifts, and shift work.	1	n/a	Dichotomous
	Company size	Asking about the size of the company with answer options 1–9, 10–99 and ≥ 100	1	n/a	Categorical
	Company sector	Asking participants about their company sector, with 13 answer options (depicting the most common occupational sectors in the Netherlands) and the answer option ‘other’	1	n/a	Categorical
	Time since first day of sick leave	Asking participants about their day of sick listing, from which the number of days between the first day of sick leave and enrolment in the study can be calculated	1	1	Continuous
	Employment status partner (if applicable)	Asking participants whether their partner has a job (full-time or part-time), with answer option yes/no	1	n/a	Dichotomous
	Caregiving leave	Asking participants whether they take caregiver leave (yes/no), and if so, for how many hours per week.	2	n/a	Dichotomous and continuous
Work	Social support from supervisor/colleagues	Two subscales of the validated Dutch version of the Job Content Questionnaire (JCQ [38];), with four items each (range 4–16).	8	4 to 16 (per subscale)	Continuous
	Job insecurity	One item (four point scale) of the Dutch Questionnaire on Perception and Judgment of Work (VBBA [39];).	1	4-point scale	Continuous
	Need for recovery	11-items (two point scale) of the VBBA [39].	11	0 to 22	Continuous
	Work accommodation	Structural changes in the job/function, and the contracted working hours, but also regarding offered and accepted accommodations, e.g., lighter work, reduced hours, modified tasks, more breaks, flexible schedule.	9	n/a	Categorical
	Impact of COVID-19 on work	Five self-established questions asking participants about the impact of COVID on their work.	5	n/a	Categorical
	Work attitude	Dutch version of the Work Involvement Scale [40]. The scale consists of six statements regarding work and working in general, that are all scored on a 7-point scale. A sample item is: ‘Having a job is very important to me.’	6	0–36	Continuous
	Self-efficacy	The Dutch version of the 12-item General Self-Efficacy scale [41] (range 12–60). A sample item is: ‘When I have decided to do something, I will definitely do it.’ The Dutch version consists of 10 items.	10	11–60	Continuous
	Work-family balance	Five questions (four point scale) from the Copenhagen Psychosocial Questionnaire (COPSOQ II [42];). A higher score indicates more work-family imbalance.	5	4-point scale	Continuous
	Work intention	One question: ‘In your estimation, what are the chances that you will be at work in 6 months?’, measured with a 10-point rating scale from 1 (no chance) to 10 (very high chance).	1	10-point scale	Continuous
	Fear of COVID-19	A Dutch translation (provided by our research group) of the fear of coronavirus-19 scale [43]. The scale consists of 7 items and is scored on a 5-point rating scale, ranging from 1 (strongly disagree) to 5 (strongly agree). A higher score indicates higher fear of COVID-19.	7	7–35	Continuous
	Expectations regarding return to work	Two questions indexing whether they have a return date in mind (yes/no), and if so, what that date is.	2	n/a	Dichotomous / continuous
Economic evaluation	Health care consumption	Asking which health care professionals were visited and which medication was taken, using the iMCQ questionnaire.	19	n/a	Continuous
	Productivity loss	Asking about productivity loss, using the iPCQ questionnaire.	Varying	n/a	Continuous
	Quality of life/utility	Asking about the quality of life, using the EQ-5D questionnaire (EuroQol-5D-5L [44])	Varying	n/a	Continuous

#### RCT among cancer survivors

The primary outcome (i.e., working hours per week) will be assessed by asking cancer survivors about their current number of actual working hours. Hence, the primary outcome ranges from zero hours (for those on full sick leave) up to the number of working hours per week in the participant’s employment contract (for those fully returned to their work). This outcome was chosen as, according to the Dutch system, RTW is typically characterised by a gradual increase in work hours, while this measure is also important for workers trying to retain work. Secondary outcomes measures include: change in working hours (as a percentage of the contract hours at baseline), employment status (at work/not at work), sickness absence days, RRTW, work ability, health-related work functioning (only for cancer survivors who returned to work), time to RTW (only for cancer survivors who were on sick leave at baseline), and health-related quality of life. In addition to abovementioned outcome measures, information on a number of additional parameters (see Table 1 for a full overview) will be gathered, to describe the study sample, to include as potential confounder(s), and/or to conduct subgroup analyses.

#### Cohort study among partners of cancer survivors

The primary outcomes for partners are working hours per week and health-related quality of life. Secondary outcomes include change in working hours (as a percentage of the contract hours at baseline), ‘employment status’ (at work/not at work), sick leave, health-related work functioning, caregiver burden and depression. Information on a number of additional parameters (see Table 1 for a full overview) will be gathered to describe the study sample and/or as predictors in the prediction model.

### Statistical analyses

For hardcopy questionnaires, data entry will be verified by a 10% double data entry check. In preliminary analyses, for both the RCT and cohort study, item frequencies and missing data will be examined. Patterns of missing data will be assessed to determine non-ignorable dropout. Scores for the included scales will be calculated according to established scoring algorithms (as described in more details in Table 2). All analyses will be performed using SPSS 22.0 [45], and a two-tailed significance level of 0.05 will be considered statistically significant.

#### RCT among cancer survivors

Representativeness of participants will be evaluated by comparing cancer survivors who are included in the RCT with aggregated data of all potentially eligible cancer survivors from the NCR, using students’ t-test or appropriate non-parametric tests for the following characteristics: gender, age, time of diagnosis, tumour type and stadium and treatment. Moreover, reason of non-response at baseline (from the answer card) and during the follow-up period will be tabulated to get a better understanding of the representativeness of the study sample.

Baseline sociodemographic and clinical characteristics of the intervention group and the control group will be described and compared. Primarily, all analyses will be conducted on an intention-to-treat basis. In addition, per-protocol analyses will be carried out, in which only cancer survivors who fully complete the intervention according to protocol (i.e., having completed a minimum of three sessions: an introductory session, at least one one-on-one session with an occupational therapist, and at least one session with the reintegration consultant) will be compared to the control group.

To evaluate between-group differences on the aforementioned primary and secondary outcomes, generalized estimating equations (GEE) for longitudinal data (i.e., with the primary and secondary outcomes at six (T1) and 12 months of follow-up (T2)) will be used, adjusted for baseline values of the outcome and using appropriate tests for ordered and continuous data. This approach accounts for correlated within-subject responses, allows for non-normally distributed variables and deals adequately with missing data [46–48]. If the data are not missing completely at random, non-ignorable drop-out will be adjusted for [49]. Short- (T1) and long-term (T2) effects of the intervention will be reported separately. Effect sizes will be expressed in beta, with 95% confidence interval.

The secondary outcome ‘time to RTW’ will be analysed with time-to-event analysis [50]. A Kaplan-Meier curve will be drawn, and differences between the two groups will be tested with the log rank test. In addition, the Cox proportional hazard model of survival analysis will be applied to estimate hazard ratios and the corresponding 95% confidence intervals.

For all analyses, unadjusted models and models adjusted for relevant confounders will be presented. In addition to the main analysis described above, subgroup analyses will be performed (see Table 1 for a list of potential confounders and subgroups). No interim analyses will be conducted for this study, as due to the low risk of the intervention it is unlikely that results of such analyses would lead to termination of the study.

#### Cohort study among partners of cancer survivors

Reason of non-response before baseline (from the answer card) and during the follow-up period will be tabulated to get a better understanding of the representativeness of our sample. The distribution of baseline values of primary and secondary outcomes and additional variables will be described. The longitudinal nature of the data enables examination of the course of primary and secondary outcome measures over time in partners, using GEE for longitudinal data with time as independent variable and the outcome variables as dependent variables.

A time lag prediction model will be used to determine which characteristics predict the primary outcomes in partners of cancer survivors [48]. In this time lag model, the measurements of the predictors will be related to outcomes status 6 months later, thus relating baseline (T0) predictors to outcomes at T1, and predictors at T1 to outcomes at T2 [51]. Univariate analyses with *p* < 0.10 will be conducted, to select possible relevant predictors. Continued selection on the remaining predictors from the univariate analyses will be based on multi-collinearity and accepted in the multivariate GEE analyses if correlation coefficients are ≥ − 0.7 and ≤ 0.7 [52].

### Process evaluation RCT

Alongside the RCT, a process evaluation will be conducted using the RE-AIM (Reach, Effectiveness, Adoption, Implementation, and Maintenance) framework [53], to examine the STEPS intervention regarding feasibility, experiences, satisfaction, barriers and facilitators for implementation. The RE-AIM framework has been adjusted to the design and target population of this study (see Table 3 for an overview), e.g., adding the dimension ‘tailoring’ to the framework. The measurements of the process evaluation will be based on data collected during the study and data collected by process evaluation questionnaires. To do so, cancer survivors in the intervention group will receive a short questionnaire, together with the six-month follow-up questionnaire (T1). Cancer survivors in the control group will be asked what care they have received during the study period. Participating occupational therapists and reintegration consultants will receive their own specified questionnaire at the end of the intervention period.

**Table 3:** Overview of process measures according to the Re-Aim framework

Dimensions	Topics	Assessment method
Reach (individual level)	1. Patient response versus nonresponse rate. 2. Characteristics of participants compared to non-participants. 3. Percentage of patients who completed the intervention.	1. Response and non-response data. 2. Percentages excluded and reasons for exclusion (using drop-out and loss-to-follow-up data, reported reasons for declining participation, data from NCR on source population). 3. Process evaluation questionnaire (patient version).
Effectiveness (individual level)	4. Effect of the intervention on primary and secondary outcomes. 5. Unintended adverse effects of the program.	4. Data regarding primary and secondary outcome measures. 5. Self-reported in process evaluation questionnaire.
Tailoring (individual level)	6. Extent to which the content, intensity, and duration of the intervention was tailored to the patient’s needs, limitations, wishes and capacities. 7. Extent to which the intervention enabled the patient to return to work or continue work. 8. Extent to which the return-to-work/work retention plan fitted the work-related needs, capacities and wishes of the patient.	6–8. Process evaluation questionnaire (patient version).
Adoption (organizational level)	9. Attitude from the occupational therapists and reintegration consultants regarding the intervention after adoption of the program. 10. Extent to which the program corresponded with organizational goals and capacities of the participating centres hospital and the reintegration agency.	9. Process evaluation questionnaire (occupational therapist and reintegration consultant version). 10. Data regarding (cost-)effectiveness of the intervention and data from the process evaluation questionnaire (occupational therapist and reintegration consultant version).
Implementation (individual and organizational level)	11. The extent to which recruitment was conducted according to protocol. 12. The extent to which the intervention was delivered according to protocol. 13. Extent to which the intervention was delivered according to budget.	11. Evaluating through what recruitment path (via the NCR, health care practitioners or social media) 12. Process evaluation questionnaire (occupational therapist and reintegration consultant version). 13. Measured by evaluating study procedures.
Maintenance (individual, organizational level)	14. Extent to which the program produced long-term individual behaviour change and established return to work. 15. Extent to which organizations will sustain the program in the future. 16. Extent to which future patients are likely to participate in the program.	14. Data regarding the primary and secondary outcome measures. 15. Process evaluation questionnaire (occupational therapists and reintegration consultant version). 16. Process evaluation questionnaire (patient version).

### Economic evaluation RCT

An economic evaluation will be conducted using the societal perspective, alongside the RCT, to assess the cost-effectiveness and cost-utility of the STEPS intervention compared to usual care. The costs will be evaluated with: 1) medical consumption costs (direct health care and direct non-health care costs, e.g. taxi costs obtained from the study declarations), 2) costs related to productivity loss (indirect costs) for both the intervention and the control group, and 3) the costs to deliver the intervention program. The effectiveness will be evaluated with: 1) working hours as measured in the trial (primary outcome), and 2) the utility expressed in quality of adjusted life years (QALYs) [54].

Medical consumption costs will be measured using (a selection of questions from) the iMTA’s medical consumption questionnaire for costs outside the hospital (iMCQ) (e.g., home care, informal help) [55]. Standard costs for the Netherlands [54] and prices of prescribed drugs of the Royal Dutch Society for Pharmacy [56] will be used. Costs related to productivity loss will be measured with (a selection of questions from) the iMTA’s productivity costs questionnaire (iPCQ) [55, 57]. To calculate lost productivity due to sick leave, the net number of days on sick leave will be multiplied by the estimated price of production loss of a worker per day of sick leave, based on age and gender. In case of partial sick leave, it will be assumed that cancer survivors were 100% productive during the hours of partial work resumption. The cumulative net number of days of sick leave will be converted into working hour equivalents based on a Dutch average of 1540 working hours per year, according to the Dutch guidelines [54]. To calculate these costs, the friction cost method will be used, which captures lost productivity costs only until an employee would likely be replaced by someone currently unemployed [58]. Both iMCQ and iPCQ questions will be added to the baseline (T0) and follow-up (T1 and T2) questionnaires.

Costs to deliver the intervention will be determined by combining the training costs of the involved occupational therapists and reintegration consultants and the costs to deliver the intervention. Training costs consist of trainer costs (i.e., the expert in the preferred technique), study material costs, and attendance costs for the professionals. Costs to deliver the intervention consist of the mean hours of investment multiplied by the average wage of occupational therapists/reintegration consultant and subsequently multiplied by overhead costs. Also printing costs for the study materials will be considered. All these costs will be calculated according to the Activity Based Costing method [59], in combination with Dutch reference prices [54].

QALYs will be measured based on the utilities from the EuroQol-5D-5L [44], which will be added to the baseline and follow-up questionnaires. The Dutch tariff will be used to estimate the utility of health states described by cancer survivors. QALYs will be calculated by multiplying the utility with the amount of time a cancer survivor spend in a particular health state.

For both cost-utility (expressed in cost/QALY) and cost-effectiveness (expressed in cost/additional working hour) analyses, incremental cost-effectiveness ratios will be calculated by dividing the incremental costs by the incremental effects. The incremental cost-utility ratio indicates the additional costs needed to gain one extra QALY; the incremental cost-effectiveness ratio indicates the additional costs needed for the intervention to gain one extra unit of effect (in our case the primary outcome: additional working hour) compared to usual care. Uncertainty surrounding the input parameters will be estimated using non-parametric bootstrapping with 5000 replications [60]. The 95% confidence intervals around the mean differences will be estimated using the approximate bootstrap confidence algorithm [61]. Bootstrapped cost-effect pairs will be plotted on a cost-effectiveness plane, and a cost-effectiveness acceptability curve will be estimated applying the willingness to pay threshold [62]. The robustness of the model will be tested using various (one-way and two-way) sensitivity analyses.

### Project management

Findings from this study will be reported in scientific journal articles. The current study team will be leading authors on these articles, possibly supplemented by representatives from participating hospitals and only if Vancouver publication guidelines will be met.

Pseudonymous participant data will be stored on secured Amsterdam UMC servers, which are only accessible for the researchers. Keys to identify participants will be locked in a separate location. Regular monitoring of the data and procedures followed will be done in accordance to Dutch legislation for medical research by an independent monitor and in accordance to the monitoring plan developed by the researchers. We strive to make study data available for future research upon reasonable request.

## Results

Recruitment of both the RCT and cohort study is planned to start in September 2021 and will run until approximately September 2022. Data collection will be completed once the final participant has finalized the last 12-month follow-up questionnaire (approximately September 2023). Data analysis will commence approximately March 2023. It is aimed to finalize the study by September 2023.

## Discussion

In this paper, the rationale for the STEPS study has been described consisting of: 1) a multi-centre RCT to assess the (cost-)effectiveness of the STEPS intervention, a multidisciplinary, Stages of Change-based intervention combining occupational therapy and reintegration consultation to support RTW and work retention in cancer survivors, and 2) a prospective cohort study to assess work- and health-related outcomes in partners of cancer survivors and to study which factors predict these outcomes. Regarding the RCT, it is hypothesised that the STEPS intervention, compared to usual care, will be cost-effective and effective regarding the primary and secondary health and work-related outcome measures. The extra costs of the STEPS program likely outweigh against the gains in reduced productivity loss. If proven to be (cost-)effective, the STEPS intervention will be a valuable addition to standard care for cancer survivors, which currently varies greatly in the extent to which work-related issues are addressed [4]. Results from this RCT will also help to improve the work-related care of cancer survivors as it will provide insights into which elements of work-related care are effective and which elements are not. Regarding the cohort study, it is hypothesised that living with a cancer survivor will have a substantial negative impact on work- and health-related outcomes of the partners of these survivors. Moreover, factors that that predict the development of these outcomes are expected to be identified. This information can be used to better tailor support for cancer survivors’ partners.

### Methodological considerations

The STEPS intervention is targeted to improve various shortcomings of current interventions to support sustained employment in cancer survivors, and to improve the synergy between relevant stakeholders within the work-related care available to cancer survivors in the Netherlands. The STEPS program was designed both bottom-up (i.e., theory-driven, using the stages of change model [11]) and top-down (i.e., by interviews with experts and cancer survivors, as described in more detail elsewhere (Zegers AD, Coenen P, Bültmann, van de Poll-Franse LV, van der Beek AJ, Duijts SFA. Tailoring work participation support for cancer survivors using the Readiness for Return to Work scale: perspectives and opinions of (health care) professionals and cancer survivors. In preparation.)). This mix of a theoretical foundation that is challenged and added onto by experts and cancer survivors provides a strong basis upon which the STEPS intervention components were built.

The STEPS program will be tested in a high-quality RCT with a primary outcome measure that lends itself to between-study comparisons thanks to its simplicity (i.e., number of working hours per week). However, this outcome measure might suggest that any increase is desirable whereas, depending on the cancer survivors’ personal circumstances, this might not be the case for every cancer survivor. Thus, a particular increase in working hours per week may mean something else for a cancer survivor who has a full-time employment contract compared to a cancer survivor with a part-time contract. To accommodate this, we differentiate actual working hours per week from change in working hours (in %).

STEPS is aimed at cancer survivors who are on full sick leave (but who have an employment contract) up to those who are fully back at work, and provides assistance at 3–18 months post-diagnosis. This way, the STEPS study contributes to the currently scant evidence base on interventions for supporting work retention in cancer survivors. Additionally, STEPS is designed to be inclusive of various cancer diagnoses. It is known from the scientific literature that engaging cancer survivors in work-related conversations in the hospital setting can be beneficial in terms of RTW and/or work retention [63]. Moreover, cancer survivors, albeit sometimes retroactively, express a need for such a work-related conversation early on in the illness trajectory [16, 64]. Earlier evidence has suggested that Dutch health care providers may initiate these conversations in a slightly biased manner (i.e., selectively based on age, tumour type, and gender) [4]. Due to its inclusiveness, STEPS might reduce some of these biases.

Further, STEPS is a multidisciplinary rehabilitation program, consisting of occupational therapy and reintegration consultation. It is known from the literature that multidisciplinary programs generally produce higher effect-sizes in terms of RTW than unitary interventions [6]. Simultaneously, we are cautious not to overburden cancer survivors by not including more than two inter-related, complementary components within STEPS [9]. An important strength of the STEPS program is its potential to engender synergy between various parties involved in the sustained employment process for cancer survivors. In the Netherlands, this collaboration often lacks, in part, due to the partition between general and occupational health care.

A last strength of the STEPS study is its attention to the work- and health-related outcomes of partners of cancer survivors. More evidence on this topic can provide directions for guidelines to support this understudied group.

Several limitations of the current study should be mentioned. To start, due to the nature of the intervention and usual care, it is not possible to perform blinding in the RCT. It is conceivable that cancer survivors in the control group might exert more effort in seeking out appropriate care within usual care than cancer survivors in usual care typically would, due to their awareness of their allocation in the control group. This might obscure the effects of the STEPS program. Our process evaluation including an inventory among the control group may provide insight and help interpret the findings.

Recruitment difficulties are expected to occur, due to our aim of recruiting cancer survivors 3–18 months post-diagnosis. Although this early timeframe was approved by various experts, cancer survivors and the medical ethical committee (Zegers AD, Coenen P, Bültmann, van de Poll-Franse LV, van der Beek AJ, Duijts SFA. Tailoring work participation support for cancer survivors using the Readiness for Return to Work scale: perspectives and opinions of (health care) professionals and cancer survivors. In preparation.), it is possible that this innovative element might result in underrepresentation of survivors, as cancer survivors might not be open to a rehabilitation program relatively early after diagnosis. It is aimed to prevent this by establishing good communication with surgeons and other health care professionals who are participating in our study, so that they can apprise cancer survivors of the benefits of early participation in work-related interventions. These benefits will also be communicated in our invitation letter and information brochure, and emphasize that cancer survivors can participate, even though they do not feel ready to RTW at time of invitation. It is further emphasized that the STEPS program is tailored to fit cancer survivors’ needs, and that they will not be pressured to RTW by the intervention providers.

In the RCT, cancer survivors who are entrepreneurs or are engaged in other work outside of contractual employment will not be included. This selection was made to assess, for instance, the effects of supporting communication between cancer survivors and their employers on sustained employment. Another reason for excluding entrepreneurs is that these self-employed people have a different safety net where RTW or work retention will be different from that of someone who is working for an employer. Also, cancer survivors who are unwilling to involve their employer in the intervention will be excluded, which may bias our sample. Lastly, cancer survivors will be excluded if they have only received surgery or any other treatment without additionally receiving chemo- and/or radiotherapy. It is known that chemo- and/or radiotherapy produce more adverse and long-term effects, affecting work ability, than surgery only. Nonetheless, STEPS could still be beneficial for cancer survivors who fall outside of the scope of the current RCT.

## Conclusion

The design of the STEPS study has been described consisting of: a multi-centre RCT assessing the (cost-)effectiveness of the STEPS intervention, and a prospective cohort study to assess work- and health-related outcomes in partners of cancer survivors and to study which factors predict these outcomes. Results from the RCT will help to improve the work-related care of cancer survivors, while information from the cohort study can be used to better tailor support for cancer survivors’ partners.

## Supplementary Information

**Additional file 1.**

**Additional file 2.**

## Data Availability

Not applicable. No data are presented in this article.

## Abbreviations

RTW: Return to work
RCT: Randomized controlled trial
STEPS: SusTained Employability in cancer Patients and their partnerS
SPIRIT: Standard protocol items for clinical trials
NCR: Netherlands Cancer Registry
NFK: Cancer Patient Organizations
IPSO: Psychosocial oncology walk-in centres [Instellingen PsychoSociale Oncologie]
GCP: Good Clinical Practice
RRTW: Readiness for Return To Work
GEE: Generalized estimating eqs.
RE-AIM: Reach, Effectiveness, Adoption, Implementation, and Maintenance
QALYs: Quality of adjusted life years
