# Supplementary material for: Supporting participation in paid work of cancer survivors and their partners in the Netherlands: protocol of the SusTained Employability in cancer Patients and their partnerS (STEPS) multi-centre randomized controlled trial and cohort study
Source: BMC Public Health. 2021 Oct 12;21:1844. doi: 10.1186/s12889-021-11865-8 (PMC8506084; doi:10.1186/s12889-021-11865-8)
Supplement: Supplementary file 1 — Additional file 1. [file 12889_2021_11865_MOESM1_ESM.docx]

Supplementary file 1. List of participating hospitals in the STEPS study.

- Amsterdam UMC, Amsterdam
- Maxima Medical Center, Eindhoven
- Dijklander Hospital, Hoorn
- Antoni van Leeuwenhoek Hospital, Amsterdam
- Haaglanden Medical Center, The Hague
- Van Weel-Bethesda Hospital, Dirksland
