# Supplementary material for: Supporting participation in paid work of cancer survivors and their partners in the Netherlands: protocol of the SusTained Employability in cancer Patients and their partnerS (STEPS) multi-centre randomized controlled trial and cohort study
Source: BMC Public Health. 2021 Oct 12;21:1844. doi: 10.1186/s12889-021-11865-8 (PMC8506084; doi:10.1186/s12889-021-11865-8)
Supplement: Supplementary file 2 — Additional file 2. [file 12889_2021_11865_MOESM2_ESM.docx]

Supplementary file 2. STEPS intervention content per behavioural change stage.

**Possible conversational elements Tools (from handbook)**

Indexing relevant medical, personal, and work-related aspects

Asking about current and future barriers and facilitators to RTW^†^/work retention

Agenda mapping and goal setting for follow-up meetings Goal setting for RTW/work retention

Encouraging participant to initiate/keep in contact with employer

Green Motivational interviewing^‡^: responding to sustain talk

Motivational interviewing: evoking change talk

Motivational interviewing: looking forward to future RTW

Encouraging participant to initiate/keep in contact with employer (all traffic light colors)

Orange What is the participant’s status quo: describing a regular day

TTM^§^ change processes: dramatic relief, allowing grief and emotional processing
over what has happened and what has been lost or altered in the participant’s life

TTM change processes: consciousness raising, gaining understanding in one’s Info session with re-integration consultant
circumstances regarding work and what is legally required

Weighing pros and cons of future RTW Values and work roles task

Red STEPS intervision^¶^ to discuss cases

Green Motivational interviewing: evoking change talk

Motivational interviewing: responding to sustain talk and dishonest change talk

TTM change processes: consciousness raising (see Pre-contemplation) Info session with re-integration consultant

Encouraging participant to initiate/keep in contact with employer (all traffic light colors)

Orange TTM change processes: self-reevaluation (value clarification) Values and work roles task

Motivational interviewing: responding to ambiguity and affirming effort

Red STEPS intervision to discuss cases

**Introductory meeting**

**Follow-up meetings**

Pre-contemplation

*I am not yet thinking about RTW.*

Contemplation

*I am starting to consider RTW.*


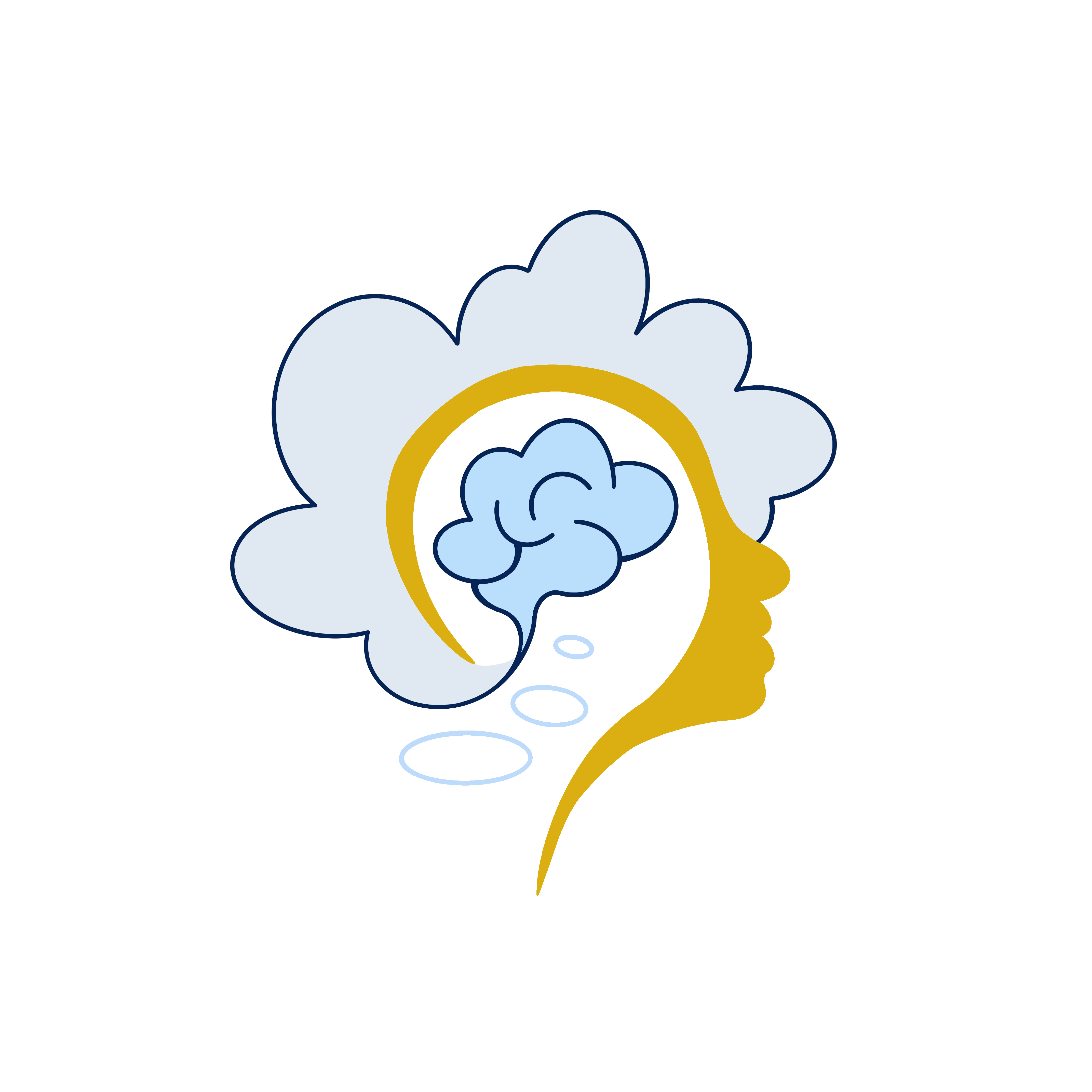


Preparation Self-Evaluative

*I would like to RTW but I am unsure what that would look like and what my options are.*


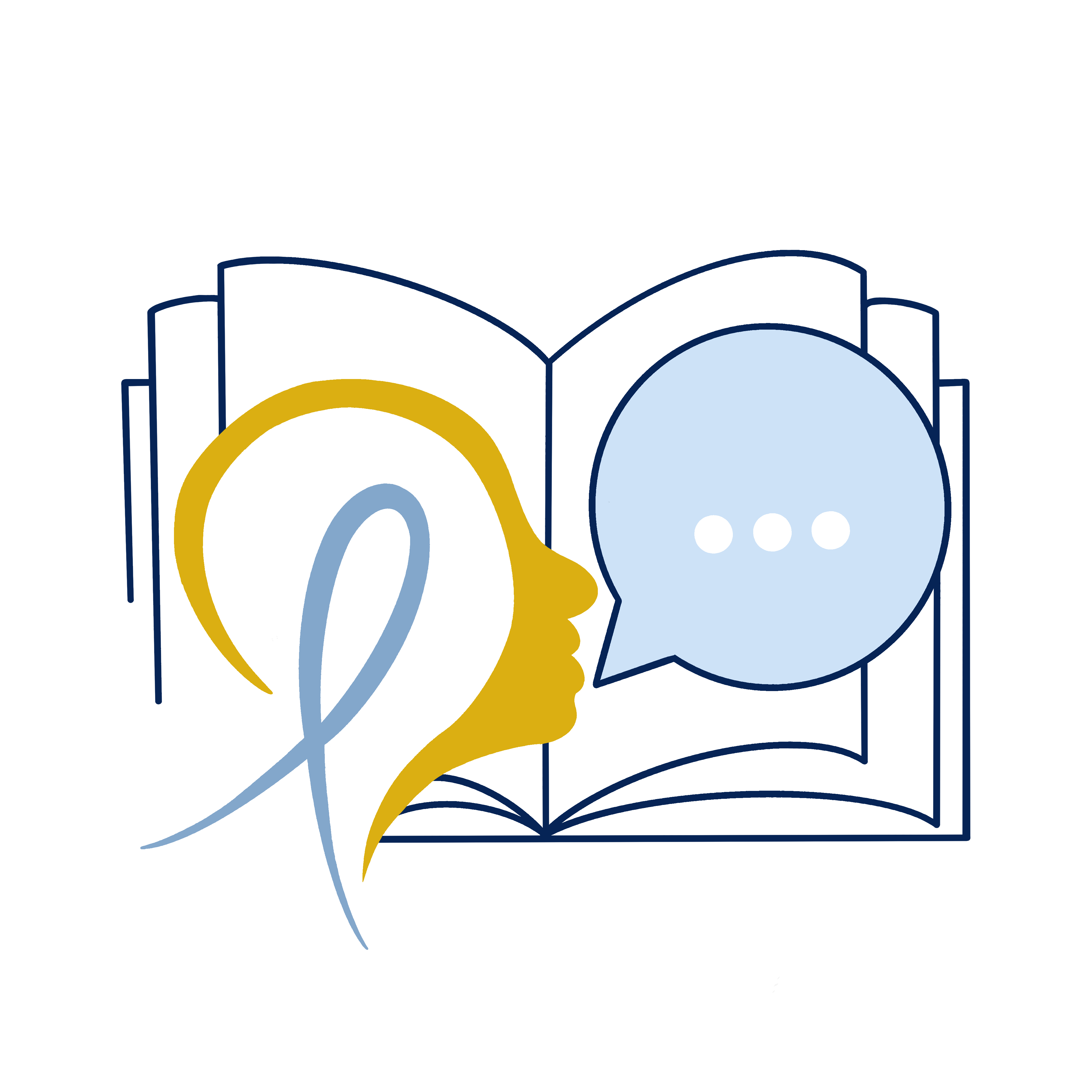


Preparation Behavioral

*I am ready to take action towards RTW.*


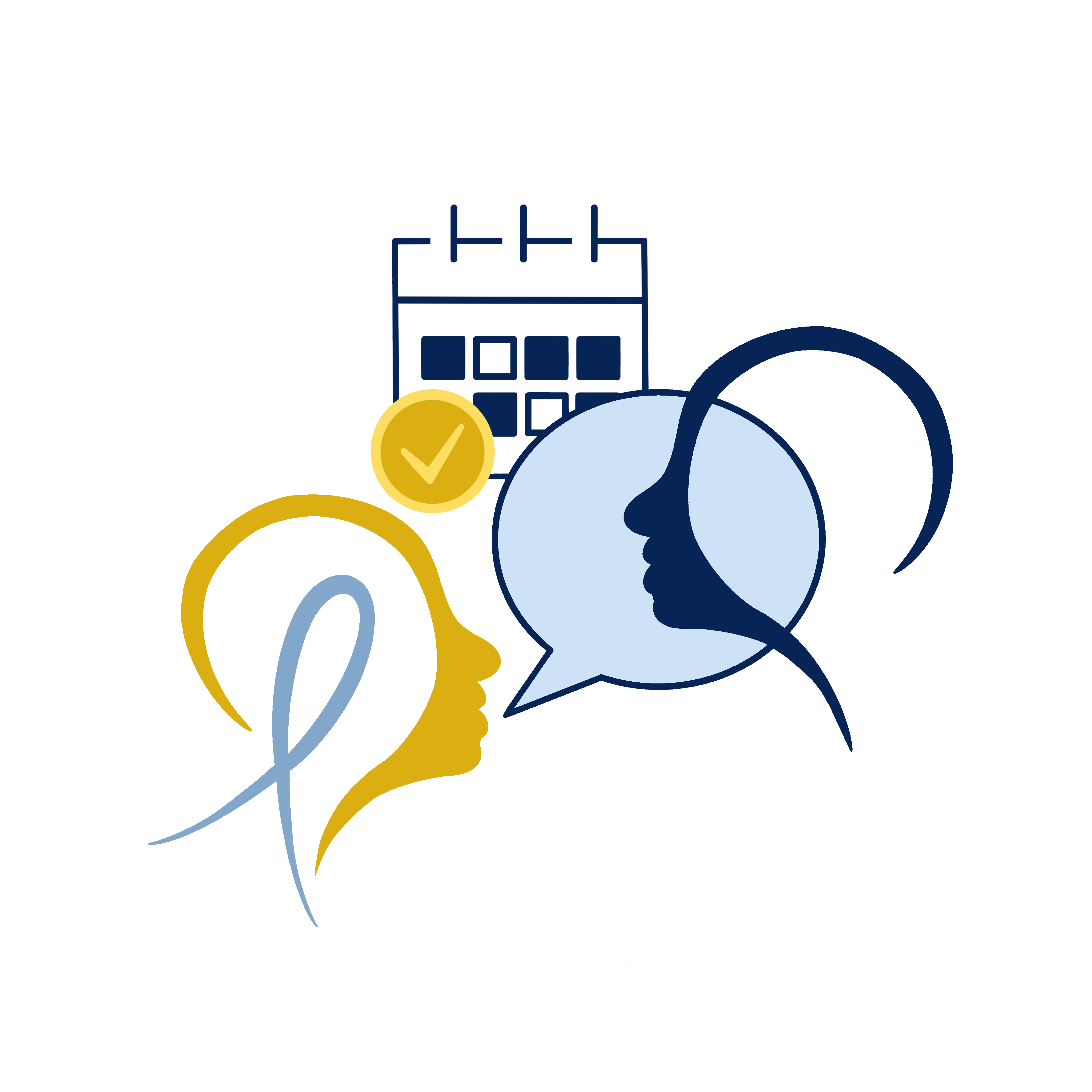


**Possible conversational elements Tools (from handbook)**

Green Reviewing and discussing occupational physician’s advice on current and future workability (if applicable)

Gaining insight into current RTW self-efficacy Energy module

Information session with the reintegration consultant (and work representative) Information sheets

Motivational interviewing: brainstorming for RTW planning

Developing an initial RTW plan RTW planning

Motivational interviewing: change and confidence ruler related to RTW plan

Motivational interviewing: evoking commitment talk and mobilizing change talk

Encouraging participant to initiate/keep in contact with employer (all traffic light colors)

Orange Motivational interviewing: looking back (strategy for evoking change talk)

Motivational interviewing: looking forward to future RTW

Motivational interviewing: reframing past failed attempts to RTW

Motivational interviewing: affirming positive attributes and effort for RTW

Develop RTW self-efficacy by training aspects like energy management Energy module

Information session with the reintegration consultant (and work representative) Information sheets

Red STEPS intervision to discuss cases

Green Motivational interviewing: affirming effort for RTW

Reviewing and discussing occupational physician’s advice on current and future workability (if applicable)

Information session with the reintegration consultant (and work representative) Information sheets

Developing an actionable RTW plan, preferably with supervisor input RTW planning

Motivational interviewing: change and confidence ruler related to RTW plan

Motivational interviewing: evoking commitment talk and mobilizing change talk

Develop RTW self-efficacy by training aspects like energy management Energy module

Putting the RTW plan in action: testing the waters and adjusting the plan RTW planning

Adjusting RTW goals if necessary Goal setting for RTW/work retention

Encouraging participant to initiate/keep in contact with employer (all traffic light colors)

Orange Responding in a supportive way to RTW setbacks

Information session with the reintegration consultant (and work representative) Information sheets

Adjusting RTW goals or RTW plan if necessary Goal setting for RTW/work retention

RTW planning

Red STEPS intervision to discuss cases

Uncertain Maintenance

*I am back at work but I am uncertain if I can sustain it.*


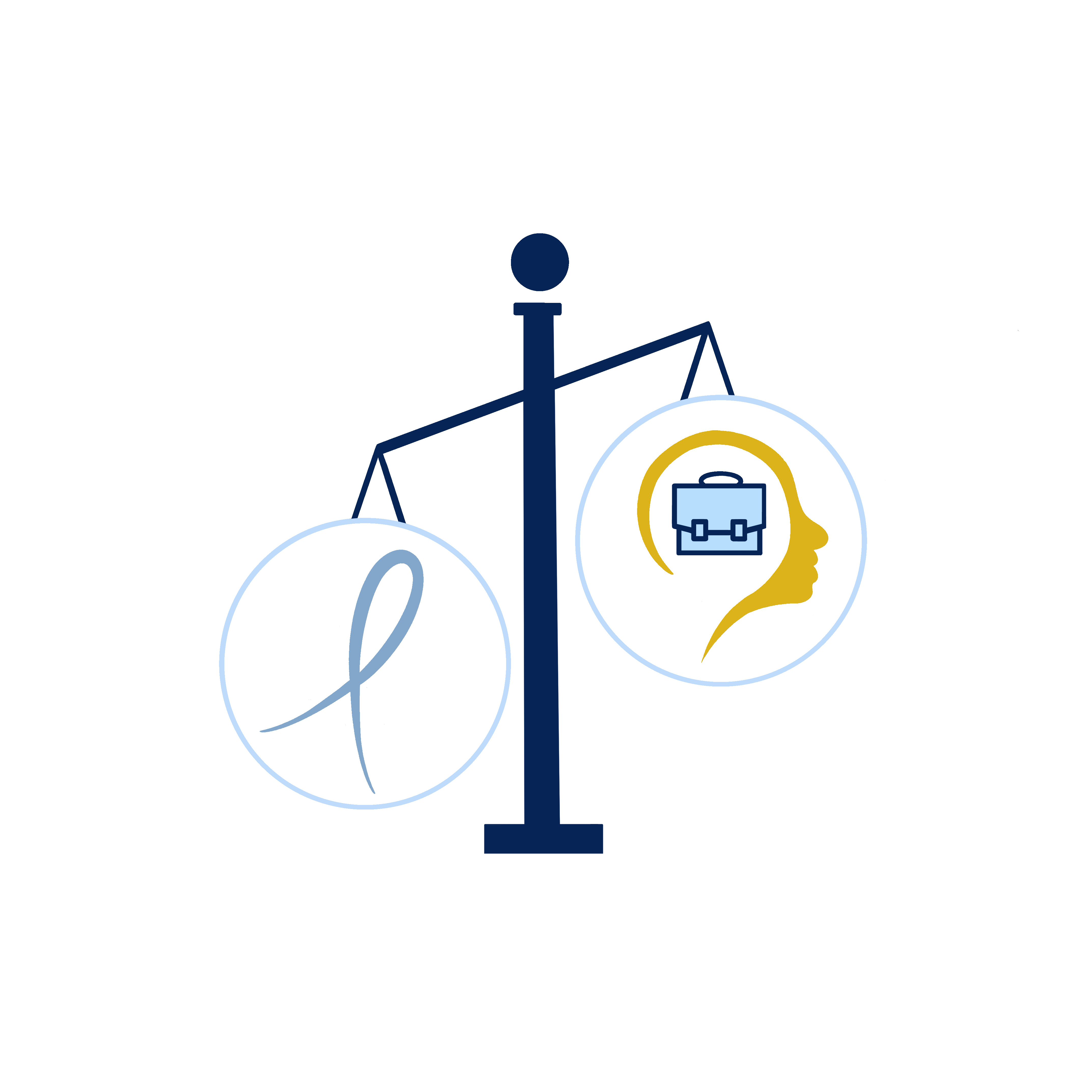


Proactive Maintenance

*I have been back at work for a while and I am confident that I can sustain it.*


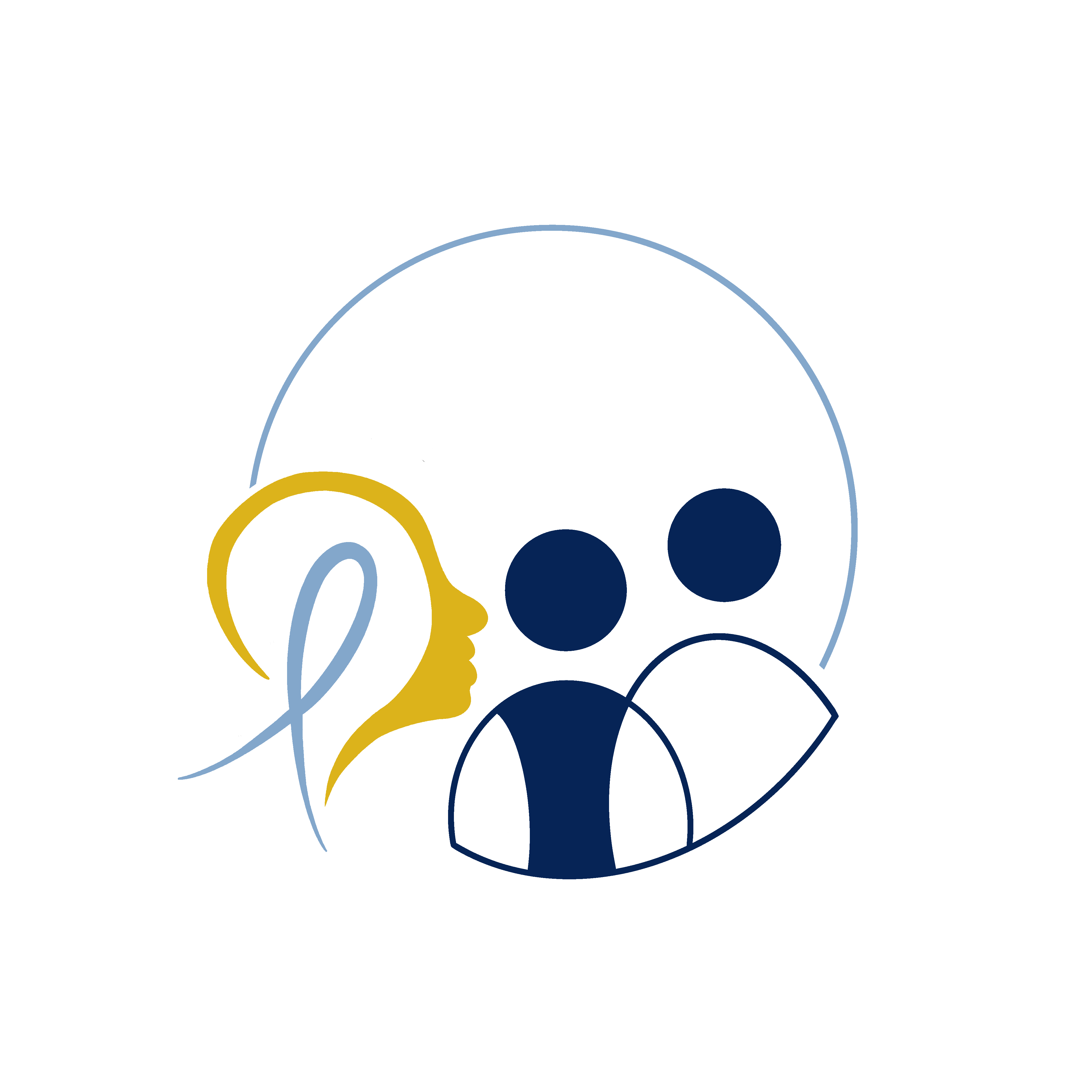


**Possible conversational elements Tools (from handbook)**

Green Developing a work retention plan Work retention planning

Information session with the reintegration consultant (and work representative) Information sheets

Develop work retention self-efficacy by training aspects like energy management Energy module

Putting the work retention plan in action: testing and adjusting the plan Work retention planning

and responding with resilience to adjustments

TTM change processes: reinforcement management by celebrating successes

Motivational interviewing: change and confidence ruler related to work retention plan

Encouraging participant to initiate/keep in contact with employer (all traffic light colors)

Orange Putting the work retention plan in action: testing and adjusting the plan Work retention planning

and responding with resilience to adjustments

Information session with the reintegration consultant (and work representative) Information sheets

Red STEPS intervision to discuss cases

Green Practical and emotional preparation for work retention (managing expectations) Work retention planning

Information session with the reintegration consultant (and work representative) Information sheets

Evaluating and adjusting the work retention plan Work retention planning

Encouraging participant to initiate/keep in contact with employer (all traffic light colors)

Orange Openly discuss motivation, barriers, and facilitators for work retention

Information session with the reintegration consultant (and work representative) Information sheets

Red STEPS intervision to discuss cases

^†^RTW = Return-To-Work; ^‡^Motivational interviewing = occupational therapists can utilize conversational strategies and techniques borrowed from motivational interviewing in the STEPS intervention. These strategies and techniques are part of a larger toolkit that occupational therapists can use in the STEPS intervention. Most occupational therapists involved in the STEPS intervention have received prior training in the basics of motivational interviewing as part of their job training. STEPS is not a motivational interviewing intervention.; ^§^TTM = TransTheoretical Model of behavior change. Change processes are the (c)overt activities that people use to progress through behavioral change stages. These natural change processes can be tapped into and thereby utilized within interventions, to help people progress through behavioral change stages faster.; ^¶^Intervision = periodic intervisions between occupational therapists involved in the intervention and the coordinating investigator, is part of the STEPS intervention. During these intervisions, complex cases will be discussed and suitable solutions will be offered by the team as a whole. Intervisions will be led by an occupational therapist with ample experience with cancer survivors returning to work.
